# Insights into the Potential Impact of Quetiapine on the Microglial Trajectory and Inflammatory Response in Organotypic Cortical Cultures Derived from Rat Offspring

**DOI:** 10.3390/biomedicines11051405

**Published:** 2023-05-09

**Authors:** Katarzyna Chamera, Katarzyna Curzytek, Kinga Kamińska, Ewa Trojan, Monika Leśkiewicz, Kinga Tylek, Magdalena Regulska, Agnieszka Basta-Kaim

**Affiliations:** Laboratory of Immunoendocrinology, Department of Experimental Neuroendocrinology, Maj Institute of Pharmacology, Polish Academy of Sciences, 12 Smętna St., 31-343 Kraków, Poland

**Keywords:** quetiapine, lipopolysaccharide, organotypic cortical cultures, maternal immune activation, CD200Fc, microglia

## Abstract

Atypical antipsychotics currently constitute the first-line medication for schizophrenia, with quetiapine being one of the most commonly prescribed representatives of the group. Along with its specific affinity for multiple receptors, this compound exerts other biological characteristics, among which anti-inflammatory effects are strongly suggested. Simultaneously, published data indicated that inflammation and microglial activation could be diminished by stimulation of the CD200 receptor (CD200R), which takes place by binding to its ligand (CD200) or soluble CD200 fusion protein (CD200Fc). Therefore, in the present study, we sought to evaluate whether quetiapine could affect certain aspects of microglial activity, including the CD200-CD200R and CX3CL1-CX3CR1 axes, which are involved in the regulation of neuron–microglia interactions, as well as the expression of selected markers of the pro- and anti-inflammatory profile of microglia (*Cd40*, *Il-1β*, *Il-6*, *Cebpb*, *Cd206*, *Arg1*, *Il-10* and *Tgf-β*). Concurrently, we examined the impact of quetiapine and CD200Fc on the IL-6 and IL-10 protein levels. The abovementioned aspects were investigated in organotypic cortical cultures (OCCs) prepared from the offspring of control rats (control OCCs) or those subjected to maternal immune activation (MIA OCCs), which is a widely implemented approach to explore schizophrenia-like disturbances in animals. The experiments were performed under basal conditions and after additional exposure to the bacterial endotoxin lipopolysaccharide (LPS), according to the “two-hit” hypothesis of schizophrenia. The results of our research revealed differences between control and MIA OCCs under basal conditions and in response to treatment with LPS in terms of lactate dehydrogenase and nitric oxide release as well as *Cd200r*, *Il-1β*, *Il-6* and *Cd206* expression. The additional stimulation with the bacterial endotoxin resulted in a notable change in the mRNA levels of pro- and anti-inflammatory microglial markers in both types of OCCs. Quetiapine diminished the influence of LPS on *Il-1β*, *Il-6*, *Cebpb* and *Arg1* expression in control OCCs as well as on IL-6 and IL-10 levels in MIA OCCs. Moreover, CD200Fc reduced the impact of the bacterial endotoxin on IL-6 production in MIA OCCs. Thus, our results demonstrated that quetiapine, as well as the stimulation of CD200R by CD200Fc, beneficially affected LPS-induced neuroimmunological changes, including microglia-related activation.

## 1. Introduction

Antipsychotics are a group of compounds widely implemented in the treatment of mental disorders with the main indication being the treatment of schizophrenia [1]. Along with their affinity to dopamine (DA) receptors, these drugs impact various other targets, including serotonin (5-HT), muscarinic, adrenergic and histamine receptors [2]. Based on differences in the mechanisms of action, antipsychotics are generally categorized into two main groups, typical (first-generation) and atypical, in which two subcategories are distinguished: second- and third-generation [3,4]. Currently, atypical antipsychotics are considered the first-line medication for schizophrenia due to numerous claims regarding their higher efficacy, safety and tolerability when compared to typical drugs [5,6].

One of the most commonly prescribed representatives of second-generation atypical antipsychotics is quetiapine. Along with a broad range of clinical utility across several neuropsychiatric disorders [7,8,9], this compound has a lower potential to produce extrapyramidal symptoms or hyperprolactinemia [10,11]. In schizophrenic patients, quetiapine has shown efficacy against both positive and negative symptoms and has benefits in improving cognitive impairment, aggression, hostility and affective disturbances [12]. The favourable action of this drug has also been described in animal models of schizophrenia-like alterations. In rats after basolateral amygdala lesions generated using quinolinic acid [13], as well as in a rodent model based on prenatal exposure to polyinosinic-polycytidylic acid [14], quetiapine normalized deficits in prepulse inhibition of sensorimotor gating. A similar phenomenon was reported for rat offspring subjected to lipopolysaccharide (LPS) in the prenatal period [15]. Furthermore, treatment with quetiapine attenuated behaviours resembling this neurological condition (e.g., hyperactivity, spatial working memory impairments and sensorimotor gating deficits) in MK-801-injected mice [16].

At the molecular level, quetiapine acts as an antagonist in various pathways, including DA transmission, 5-HT_2A_, 5-HT_2B_, 5-HT_2C_, α_1_- and α_2_-adrenergic as well as H_1_-histamine receptors [17,18]. Additionally, this drug has an affinity for the 5-HT_1A_ receptor as a partial agonist [18].

Multiple preclinical studies have demonstrated that in addition to its antipsychotic activity, quetiapine exerts other biological effects, among which its impact on the immune response is of particular interest [19,20,21,22]. It has been proven that quetiapine inhibited the release of nitric oxide (NO) and tumour necrosis factor-α (TNF-α) from interferon-γ-activated microglia *in vitro* [19], and ameliorated higher production of interleukin-6 (IL-6) and TNF-α in mice following cuprizone exposure [20]. Moreover, this antipsychotic diminished microglial cell numbers in the hippocampus and reduced Aβ-generated glial activation in a transgenic mouse model of Alzheimer’s disease [21,22].

Microglia possess a myriad of properties that make them an attractive candidate effector to maintain homeostasis in the brain, among others, by being under the control of immunomodulators such as CD200 and/or CX3CL1 [23]. Both ligands, secreted mainly by neurons, participate in the modulation of microglial activation by interacting with specific receptors (CD200R and CX3CR1, respectively), the localization of which is largely limited to microglial cells [24].

To cast more light on the potential involvement of these neuron–microglia axes, in particular CD200-CD200R, in the mechanisms of quetiapine action, we sought to evaluate whether this drug could affect the expression of the *Cd200*-*Cd200r* and *Cx3cl1*-*Cx3cr1* pairs as well as the protein levels of both receptors. Additionally, we examined the impact of quetiapine on certain aspects of inflammatory processes, including selected markers of the pro- and anti-inflammatory profile of microglial activity (*Cd40*, *Il-1β*, *Il-6*, *Cebpb*, *Cd206*, *Arg1*, *Il-10* and *Tgf-β*) and cytokine release (IL-6, IL-10). In the final stage of our research, we introduced a soluble CD200 fusion protein containing the ectodomain of CD200 bound to a murine IgG2a module (CD200Fc), which mimics the effects of the ligand CD200 [25,26], to determine whether the modulation of CD200R could influence the IL-6 and IL-10 protein levels.

Considering the specificity of our research, the study described in this article was performed using organotypic cortical cultures (OCCs), which constitute an ex vivo system preserving several aspects of the structural and synaptic organization of the original tissue, including neuron–microglia interactions [27]. Herein, we employed OCCs prepared from control rat offspring (control OCCs) and animals that were subjected to maternal immune activation (MIA OCCs) with LPS, which is one of the widely implemented approaches to explore schizophrenia-like disturbances in animals [28,29,30,31]. The experiments were performed under basal conditions and after additional exposure to the bacterial endotoxin (LPS), complying with the “two-hit” hypothesis of schizophrenia [32].

## 2. Materials and Methods

### 2.1. Animals

Adult Wistar rats were purchased from Charles River (Sulzfeld, Germany) and housed under standard conditions with a room temperature of 23 °C, 12/12 h light/dark cycle (lights on at 6:00 am) and ad libitum access to water and food. A set of pregnant females (*n* = 22) was generated as previously described [30,33] and randomly divided into two equal groups: (1) control and (2) MIA. All procedures were performed under the approval of the Animal Care Committee of the Maj Institute of Pharmacology, Polish Academy of Sciences, Cracow and followed the recommendations of the International Council for Laboratory Animal Science and Guide for the Care and Use of Laboratory Animals (consent numbers: 236/2016 and 128/2018). All possible efforts were made to minimize the number of animals used and their suffering.

### 2.2. Prenatal Exposure to LPS

MIA was induced by the administration of LPS to pregnant rats as previously reported [29,30,33,34,35]. LPS from *Escherichia coli* 026:B6 (Sigma-Aldrich, St. Louis, MO, USA) was dissolved to obtain a concentration of 2 mg/kg in 1 mL of saline. The solution was subcutaneously administered to females of the MIA group on alternate days starting from the 7th day of pregnancy between 9:00 and 10:00 a.m. Control pregnant animals underwent the same treatment regimen with the corresponding volume (1 mL/kg) of saline. After delivery, the pups were housed with dams until postnatal days 6–7 (PND6–7). No litter size or weight differences were noted between the control and MIA offspring.

### 2.3. Organotypic Cortical Cultures

Organotypic cultures were prepared based on the procedure of Stoppini et al. [36], with slight modifications, from the frontal cortices of pups at PND6–7 from both the control (control OCCs) and MIA (MIA OCCs) groups. The animals were decapitated, the brains were aseptically removed and placed in an ice-cold working buffer consisting of 96% Hanks’ balanced salt solution (HBSS) without salts, 3.5% glucose, 0.4% penicillin and streptomycin solution and HEPES (to maintain the pH) (all from Gibco, Paisley, UK). Then, the frontal cortices were dissected, transferred to Teflon discs, transversely cut into 350 μm slices using a McIlwain tissue chopper and situated on ThinCerts^TM^—TC Inserts (Greiner bio-one, Kremsmünster, Austria) with 0.4 μm pore size transparent membranes in 6-well plates. The sections were cultured with 1 mL of Dulbecco’s Modified Eagle Medium (DMEM) + GlutaMax™-I (50%; pH 7.4) supplemented with 20.5% HBSS with salts, 25% horse serum (HS), 0.1 mg/mL glucose, 1% amphotericin B, 0.4% penicillin and streptomycin solution, 1% B-27 supplement and HEPES (to maintain pH at 7.4) (all from Gibco, Paisley, UK) in a humidified 5% CO_2_ incubator at 37 °C. After 24 h, half of the medium volume (0.5 mL) was replaced, and then after the next 48 h, 1 mL of medium was changed to a fresh one. Later, the medium was substituted (1 mL) every second day with a new one containing a gradually reduced amount of HS (25% and 10%). On the 7th day of in vitro culture, the medium was replaced with a serum-free 1% N2-supplemented mixture of 50% DMEM F-12 (pH 7.4), 44% HBSS with salts, 0.1 mg/mL glucose, 1% amphotericin B, 0.4% penicillin and streptomycin solution, 1% B-27 and HEPES (to maintain pH at 7.4) (all from Gibco, Paisley, UK).

### 2.4. Chemicals and Drugs

Recombinant mouse CD200 Fc chimera protein (CD200Fc; R&D Systems, Minneapolis, MN, USA) was reconstituted in sterile phosphate-buffered saline (PBS; Sigma-Aldrich, St. Louis, MO, USA) (the final concentration in the well was 5 µg/mL) [37]. Quetiapine (Carbosynth Limited, Berkshire, UK) was dissolved in dimethyl sulfoxide (BioShop, Burlington, ON, Canada) to a 10 mM stock solution and was freshly diluted before each use in PBS (Sigma-Aldrich, St. Louis, MO, USA) (the final concentration in the well was 1, 5 or 10 µM). LPS (from *Escherichia coli* 0111:B4; Sigma-Aldrich, St. Louis, MO, USA) was prepared in PBS (Sigma-Aldrich, St. Louis, MO, USA) and the final concentration in the well was 1 μg/mL [38].

### 2.5. Treatment of the OCCs

**Figure 1:**
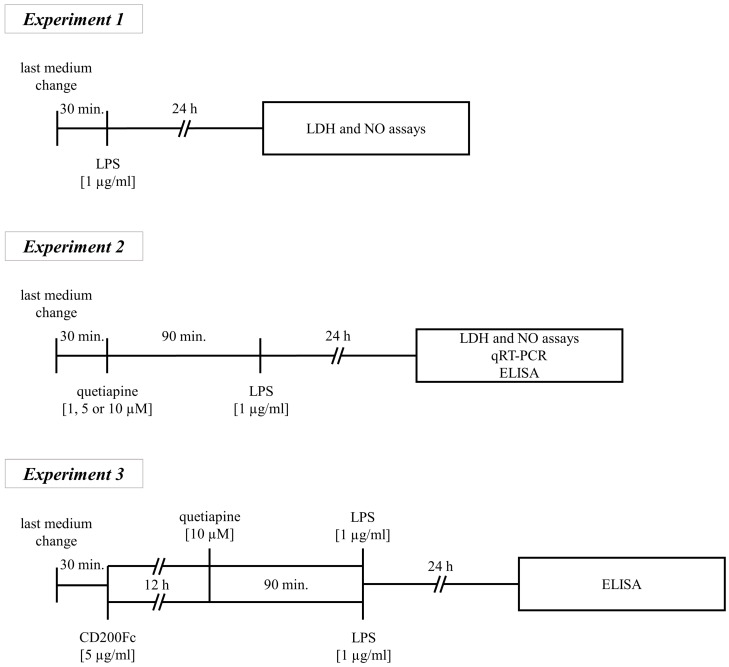
Schedule of the experimental procedure applied in the study. Thirty minutes after the last medium change, both control and MIA OCCs were subjected to one of three treatments. *Experiment 1*: OCCs were incubated with LPS (1 µg/mL) for 24 h, and LDH and NO assays were then performed. The corresponding results are presented in Figure 2. *Experiment 2*: OCCs were first exposed to quetiapine (1, 5 or 10 µM) for 90 min and later to LPS (1 µg/mL) for 24 h. LDH and NO tests (with quetiapine at all doses), ELISAs and qRT-PCR (with quetiapine at 10 µM) were carried out, and the results of these analyses are displayed in Figure 3, Figure 4, Figure 5 and Figure 6. *Experiment 3*: MIA OCCs were treated with CD200Fc (5 µg/mL) for 12 h. Quetiapine (10 µM) was added to MIA OCCs for 90 min. Then, LPS (1 µg/mL) was introduced to the medium for 24 h. The ELISA method was used, and the corresponding results are shown in Figure 7.

An overview of the treatment schedule is illustrated in Figure 1. Thirty minutes after the last medium change, control and MIA OCCs were stimulated in the manner described hereafter.

*Experiment 1*

In the experiments comparing control and MIA OCCs in terms of response to LPS exposure, the slices were subjected to this bacterial endotoxin for 24 h, and afterwards, the appropriate analyses were performed.

*Experiment 2*

In the next set of experiments determining quetiapine doses for administration in further studies and assessing the impact of the drug on mRNA expression of *Cd200*-*Cd200r* and *Cx3cl1*-*Cx3cr1* axes and markers of the pro- and anti-inflammatory profile of microglia (*Cd40*, *Il-1β*, *Il-6*, *Cebpb*, *Cd206*, *Arg1*, *Il-10* and *Tgf-β*) as well as on CD200R and CX3CR1 levels, the OCCs were incubated with the antipsychotic for 90 min and later additionally exposed to LPS for 24 h.

*Experiment 3*

In the last set of experiments comparing the potential influence of CD200Fc and quetiapine effects on MIA OCCs, the slices were treated with CD200Fc for 12 h or exposed to the drug for 90 min and finally incubated with LPS for 24 h.

Control groups in all experiments were subjected to the appropriate solvent in the corresponding volume and regimen.

### 2.6. Culture Collection and Sample Preparation

At the end of each experiment, culture media were collected for the evaluation of lactate dehydrogenase (LDH) and NO release as well as for enzyme-linked immunosorbent assay (ELISA).

Simultaneously, the slices intended for ELISA analyses were lysed in RIPA lysis buffer enriched with protease inhibitor cocktail, phosphatase inhibitor cocktail, 1 mM sodium orthovanadate and 1 mM phenylmethanesulfonyl fluoride (all from Sigma-Aldrich, St. Louis, MO, USA). The protein concentration in the prepared samples was determined utilizing a Pierce^TM^ BCA Protein Assay Kit (Thermo Fisher, Rockford, IL, USA) according to the manufacturer’s instructions. Bovine serum albumin from the kit served as a standard, and the absorbance for each sample was examined at a wavelength of 562 nm in a Tecan Infinite 200 Pro spectrophotometer (Tecan, Mannedorf, Germany).

To prepare samples for quantitative real-time polymerase chain reaction (qRT-PCR), the slices were transferred to TRI Reagent^®^ (Sigma-Aldrich, St. Louis, MO, USA), and total RNA was extracted using the Chomczynski method [39]. Instantly after extraction, RNA concentration was evaluated with a NanoDrop Spectrophotometer (ND/1000 UV/Vis, Thermo Fisher NanoDrop, Waltham, MA, USA).

### 2.7. Lactate Dehydrogenase Release

The LDH assay was performed as previously described [38] based on a colorimetric method using a commercially available Cytotoxicity Detection Kit (Roche, Mannheim, Germany) following the manufacturer’s instructions. The optical density of the red-colored solution of formazan formed during the test was measured at a wavelength of 490 nm in a Tecan Infinite 200 Pro spectrophotometer (Tecan, Mannedorf, Germany) and was proportional to the LDH level. The results were normalized to the LDH release calculated for the vehicle group from control OCCs (*Experiment 1*) or the vehicle group from the corresponding type of OCCs (*Experiment 2*) and are displayed as a percentage of the control ± standard error of the mean (SEM).

### 2.8. Nitric Oxide Release

The level of NO secreted into the culture medium was determined by the Griess reaction as previously described [38]. Equal volumes of supernatant, Griess reagent A [0.1% *N*-(1-naphthyl)ethylenediamine dihydrochloride] and Griess reagent B (1% sulfanilamide in 5% phosphoric acid) (both from Sigma-Aldrich, St. Louis, MO, USA) were mixed, and the absorbance of the prepared solution was measured immediately at a wavelength of 540 nm in a Tecan Infinite 200 Pro spectrophotometer (Tecan, Mannedorf, Germany). The data were normalized to the level of NO released from the vehicle group in control OCCs (*Experiment 1*) or the vehicle group from the corresponding type of OCCs (*Experiment 2*) and are presented as the percentage of the control ± SEM.

### 2.9. Enzyme-Linked Immunosorbent Assay

The protein levels of CD200R and CX3CR1 (both from Cusabio, Houston, TX, USA) were established in the OCC lysates, whereas levels of IL-6 and IL-10 (both from BD Biosciences, San Diego, CA, USA) were quantified in the supernatants using commercially available ELISA kits following the manufacturer’s instructions. The outcomes of ELISA experiments were calculated as pg/mg of protein or optical density at 450 nm.

### 2.10. Quantitative Real-Time Polymerase Chain Reaction

Equal amounts of RNA (0.5 μg) were reverse transcribed into complementary DNA (cDNA) employing an NG dART RT kit (EURx, Gdańsk, Poland). The cDNA was amplified with a FastStart Universal Probe Master (Rox) kit (Roche, Basel, Switzerland) and TaqMan probes (Life Technologies, Carlsbad, CA, USA) for the following genes: *Cd200r* (Rn00576646_m1), *Cd200* (Rn01646320_m1), *Cx3cr1* (Rn00591798_m1), *Cx3cl1* (Rn00593186_m1), *Cd40* (Rn01423583_m1), *Il-1β* (Rn00580432_m1), *Il-6* (Rn01410330_m1), *Cebpb* (Rn00824635_s1), *Cd206* (Rn01487342_m1), *Arg1* (Rn00691090_m1), *Il-10* (Rn01644839_m1), *Tgf-β* (Rn00572010_m1), and, as a reference, *Gapdh* (Rn01775763_g1). The PCR products were generated in mixtures consisting of cDNA (diluted 10 times in PCR grade distilled water) used as the PCR template (3 μL), TaqMan probe (0.5 μL), 1× FastStart Universal Probe Master (Rox) mix containing 250 nM hydrolysis probe labelled with the fluorescent reporter dye (fluorescein) at the 5′-end and a quenching dye at the 3′-end (5 μL), and finally, the remainder of PCR grade distilled water to reach a total volume of 10 μL. Thermocycling conditions were as follows: initial denaturation at 95 °C for 10 min, 40 cycles of denaturation at 95 °C for 15 s, annealing at 60 °C for 1 min and extension at 50 °C for 2 min. The threshold value (C_t_) for each sample was set in the exponential phase of PCR and the ∆∆C_t_ method was used for data analysis.

### 2.11. Statistical Analysis

Statistical analysis of the data was executed using Statistica 13.0 software (StatSoft, Palo Alto, CA, USA). All biochemical experiments were carried out under the same conditions, regardless of the treatment or culture (control OCCs vs. MIA OCCs). The results of all analyses are displayed as the mean ± SEM. When applicable, statistical outliers were identified using Grubbs’ test. The normal distribution and the homogeneity of the variance were examined using the Shapiro–Wilk test and Levene’s test, respectively. Comparisons of variables between groups were performed using factorial analysis of variance (factorial ANOVA) with Duncan’s post hoc test or planned comparisons via a one-way ANOVA (contrast analysis). The results were considered statistically significant when the *p* value was less than 0.05. All graphs were prepared with GraphPad Prism 7 software (San Diego, CA, USA).

## 3. Results

### 3.1. Release of Lactate Dehydrogenase and Nitric Oxide in Control and MIA OCCs under Basal and LPS-Induced Conditions

**Figure 2:**
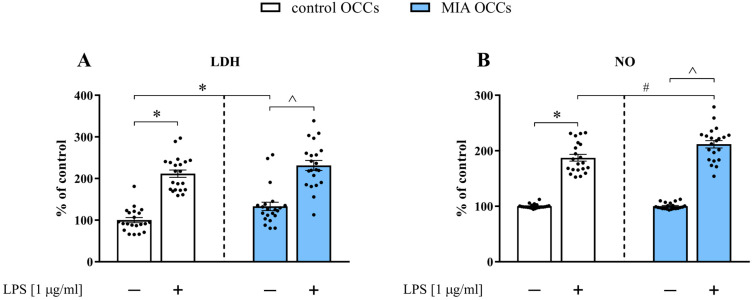
The basal and LPS-induced levels of LDH (**A**) and NO (**B**) in control and MIA OCCs. *n* = 21 in each group. The results were calculated as a percentage of the release obtained for the vehicle group from control OCCs (% of control) and are presented as the means ± standard errors of the means (SEM). Statistical analysis was performed using factorial ANOVA with Duncan’s post hoc test. * *p* < 0.05 vs. control OCCs + vehicle, # *p* < 0.05 vs. control OCCs + LPS, ^ *p* < 0.05 vs. MIA OCCs + vehicle.

In the initial experiments, we examined the basal and LPS-evoked levels of LDH and NO in OCCs obtained from control and MIA offspring (Figure 2).

As shown in Figure 2A, the nonstimulated release of LDH in MIA OCCs was significantly higher (*p* = 0.0166) than that in control OCCs. The 24-h incubation with the bacterial endotoxin resulted in the increased efflux of LDH from the slices of both control (*p* = 0.0001) and MIA (*p* = 0.0001) OCCs (Figure 2A). Regarding basal NO secretion, we did not observe any difference between the two types of OCCs (Figure 2B). Exposure to LPS elevated the level of NO in both control (*p* = 0.0001) and MIA (*p* = 0.0001) OCCs. Simultaneously, the effect of treatment with the bacterial endotoxin on NO release was more pronounced for MIA (*p* = 0.0004) than for control OCCs (Figure 2B).

### 3.2. The Impact of Different Doses of Quetiapine on Lactate Dehydrogenase and Nitric Oxide Release in Control and MIA OCCs under Basal and LPS-Induced Conditions

**Figure 3:**
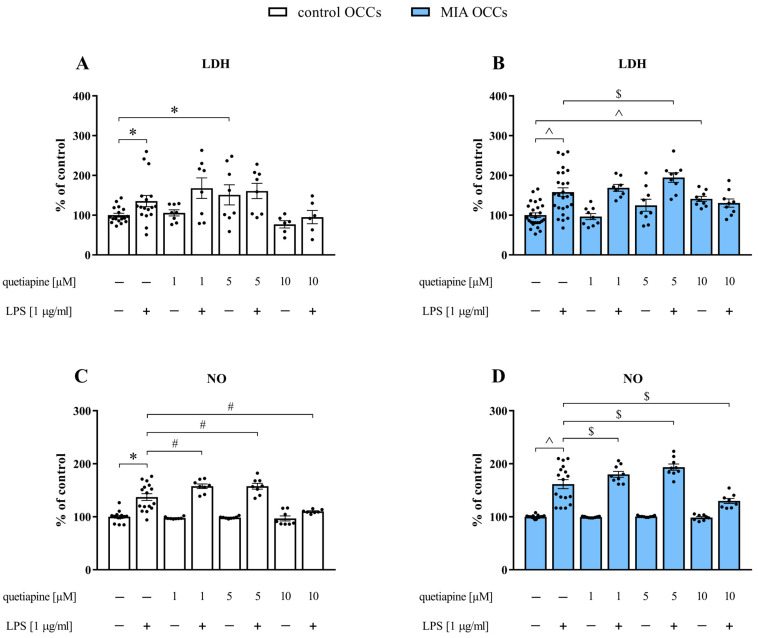
LDH (**A**,**B**) and NO (**C**,**D**) levels in control (**A**,**C**) and MIA (**B**,**D**) OCCs after treatment with different doses of quetiapine (1, 5 or 10 µM) under basal and LPS-induced conditions. *n* = 6–17 (LDH) or 7–16 (NO) in control OCCs, *n* = 8–26 (LDH) or 8–17 (NO) in MIA OCCs. The results were calculated as a percentage of the release observed for the vehicle group from the corresponding type of OCCs (% of control) and are presented as the means ± standard errors of the means (SEM). Statistical analysis was performed using planned comparisons via a one-way ANOVA (contrast analysis). * *p* < 0.05 vs. control OCCs + vehicle, # *p* < 0.05 vs. control OCCs + LPS, ^ *p* < 0.05 vs. MIA OCCs + vehicle, $ *p* < 0.05 vs. MIA OCCs + LPS.

In the next step, LDH and NO assays were applied to determine the dose of quetiapine to be used in further experiments (Figure 3).

Incubation of the slices with LPS (*p* = 0.0377), but also with the drug at a concentration of 5 μM enhanced LDH secretion in control OCCs (*p* = 0.0171) (Figure 3A). Concurrently, in MIA OCCs, stimulation with LPS (*p* < 0.0001) as well as with the antipsychotic at a dose of 10 µM (*p* = 0.0070) upregulated LDH efflux (Figure 3B). Pretreatment with 5 µM quetiapine (*p* = 0.0156) intensified LDH release in response to the addition of the bacterial endotoxin in MIA OCCs. The outcome of the NO test revealed that exposure to LPS led to the increased release of the measured compound both in control (*p* < 0.0001) and MIA (*p* < 0.0001) OCCs (Figure 3C,D). This effect was aggravated when the slices were preincubated with quetiapine at a concentration of 1 (control OCCs: *p* = 0.0021; MIA OCCs: *p* = 0.0154) or 5 µM (control OCCs: *p* = 0.0022; MIA OCCs: *p* < 0.0001) in both types of OCCs. In contrast, 10 µM quetiapine prevented the increase in NO secretion after LPS stimulation in both control (*p* = 0.0001) and MIA (*p* = 0.0001) OCCs (Figure 3C,D). Therefore, based on the above-described results showing that among the tested doses, only quetiapine at 10 µM did not enhance the effect of the bacterial endotoxin on LDH levels and at the same time reduced NO release after treatment with LPS in both types of OCCs, further experiments were carried out using the drug at a concentration of 10 µM.

### 3.3. The Impact of Quetiapine on the mRNA Expression of the Cd200-Cd200r and Cx3cl1-Cx3cr1 Axes in Control and MIA OCCs under Basal and LPS-Induced Conditions

**Figure 4:**
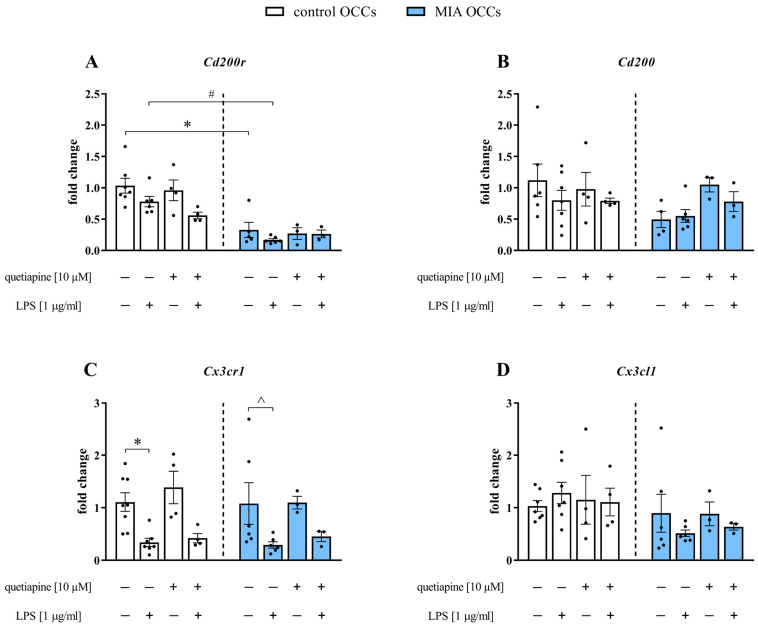
The gene expression of *Cd200r* (**A**), *Cd200* (**B**), *Cx3cr1* (**C**) and *Cx3cl1* (**D**) in control and MIA OCCs after quetiapine (10 µM) treatment under basal and LPS-induced conditions. *n* = 4–8 in control OCCs and *n* = 3–6 in MIA OCCs (*Cx3cr1*), *n* = 4–7 in control OCCs and *n* = 3–6 in MIA OCCs (*Cd200r*, *Cd200*, *Cx3cl1*). The results were calculated as the average fold change and are presented as the means ± standard errors of the means (SEM). Statistical analysis was performed using factorial ANOVA with Duncan’s post hoc test or planned comparisons via a one-way ANOVA (contrast analysis). * *p* < 0.05 vs. control OCCs + vehicle, # *p* < 0.05 vs. control OCCs + LPS, ^ *p* < 0.05 vs. MIA OCCs + vehicle.

The data suggested that microglia might be intimately engaged in MIA-triggered disturbances, resembling schizophrenia [40], and that antipsychotics may affect the activation of these cells [22]. Accordingly, we evaluated the gene expression of two microglial receptors (*Cd200r* and *Cx3cr1*) and their corresponding neuronal ligands (*Cd200* and *Cx3cl1*, respectively) in control and MIA OCCs under basal conditions and when subjected to quetiapine and/or additional LPS treatment (Figure 4).

In the case of the *Cd200*-*Cd200r* axis, we observed a lower level of the receptor (*p* = 0.0002) in MIA OCCs than in control OCCs. Simultaneously, MIA OCCs were more susceptible than control OCCs to incubation with the bacterial endotoxin in terms of *Cd200r* (*p* = 0.0010) expression (Figure 4A). When evaluating the *Cx3cl1*-*Cx3cr1* dyad, we detected a significant reduction in *Cx3cr1* mRNA levels in both control (*p* = 0.0055) and MIA (*p* = 0.0099) OCCs exposed to LPS (Figure 4C). No effect of incubation with quetiapine and/or bacterial endotoxin on the expression of the examined ligands was revealed in any of the investigated types of OCCs (Figure 4).

### 3.4. The Impact of Quetiapine on the CD200R and CX3CR1 Protein Levels in Control and MIA OCCs under Basal and LPS-Induced Conditions

**Figure 5:**
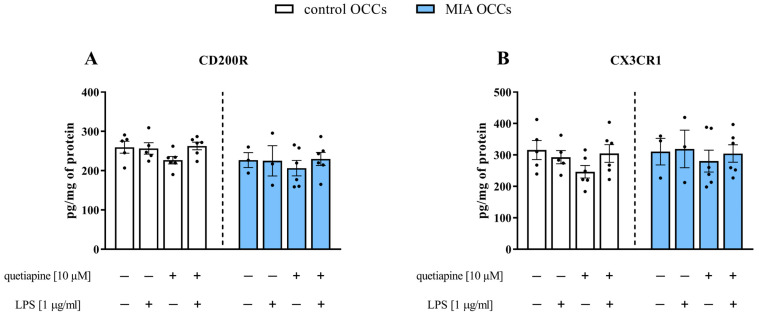
CD200R (**A**) and CX3CR1 (**B**) protein levels in control and MIA OCCs after treatment with quetiapine (10 µM) under basal and LPS-induced conditions. *n* = 3–6 in each group. The results were calculated as pg/mg of protein and are presented as the means ± standard errors of the means (SEM). Statistical analysis was performed using factorial ANOVA with Duncan’s post hoc test.

Considering the alterations in the gene expression of the systems supervising neuron–microglia interactions, in the next step of the study, we explored the protein levels of selected microglial receptors (CD200R and CX3CR1) in control and MIA OCCs after treatment with quetiapine and/or additional LPS (Figure 5). As illustrated in Figure 5, we did not notice any difference between the two types of OCCs or the impact of incubation with the drug and/or bacterial endotoxin on the levels of CD200R or CX3CR1.

### 3.5. The Impact of Quetiapine on the mRNA Expression of Microglial Markers in Control and MIA OCCs under Basal and LPS-Induced Conditions

**Figure 6:**
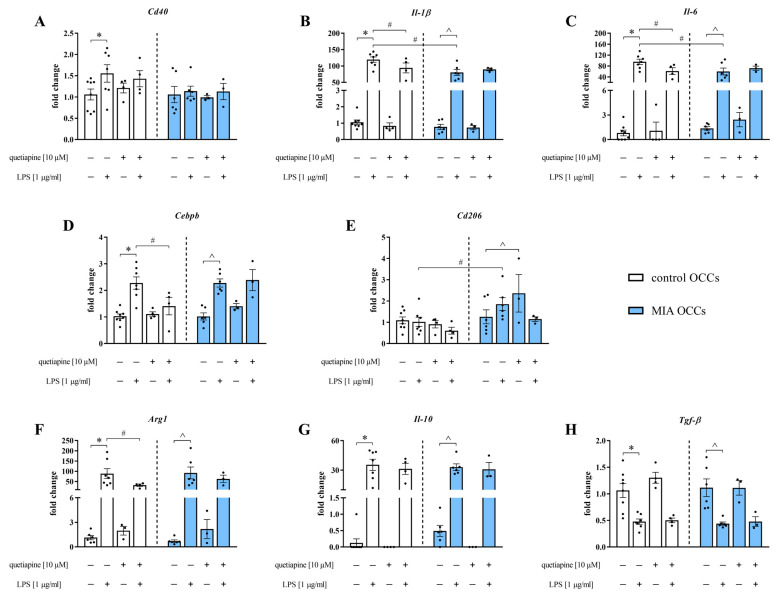
The gene expression of *Cd40* (**A**), *Il-1β* (**B**), *Il-6* (**C**), *Cebpb* (**D**), *Cd206* (**E**), *Arg1* (**F**), *Il-10* (**G**) and *Tgf-β* (**H**) in control and MIA OCCs after quetiapine (10 µM) treatment under basal and LPS-induced conditions. *n* = 4–8 in control OCCs and *n* = 3–6 in MIA OCCs (*Cd40*, *Il-1β*, *Il-6*, *Cebpb*, *Cd206*, *Il-10* and *Tgf-β*), *n* = 3–7 in control OCCs and *n* = 3–6 in MIA OCCs (*Arg1*). The results were calculated as the average fold change and are presented as the means ± standard errors of the means (SEM). Statistical analysis was performed using planned comparisons via a one-way ANOVA (contrast analysis). * *p* < 0.05 vs. control OCCs + vehicle, # *p* < 0.05 vs. control OCCs + LPS, ^ *p* < 0.05 vs. MIA OCCs + vehicle.

The response of microglia to disturbances in brain homeostasis is highly associated with the release of multiple pro- and anti-inflammatory factors as well as various CD antigen levels [41]. Therefore, we measured the expression of genes (*Cd40*, *Il-1β*, *Il-6*, *Cebpb*, *Cd206*, *Arg1*, *Il-10* and *Tgf-β*) that are considered essential for microglial activity (Figure 6).

Among the tested markers, the mRNA levels of *Cd40* (*p* = 0.0201), *Il-1β* (*p* < 0.0001), *Il-6* (*p* < 0.0001), *Cebpb* (*p* < 0.0001), *Arg1* (*p* = 0.0007) and *Il-10* (*p* < 0.0001) were increased, whereas *Tgf-β* (*p* = 0.0001) expression was decreased after LPS stimulation in control OCCs (Figure 6A–D,F–H). Pretreatment of the slices with quetiapine diminished the impact of the bacterial endotoxin on *Il-1β* (*p* = 0.0145), *Il-6* (*p* = 0.0052), *Cebpb* (*p* = 0.0031) and *Arg1* (*p* = 0.0386) levels (Figure 6B–D,F).

qRT-PCR analysis of MIA OCCs demonstrated that the expression of *Il-1β* (*p* < 0.0001), *Il-6* (*p* < 0.0001), *Cebpb* (*p* < 0.0001), *Arg1* (*p* = 0.0007) and *Il-10* (*p* < 0.0001) was elevated, and simultaneously, *Tgf-β* (*p* = 0.0001) levels were reduced in response to LPS exposure (Figure 6B–D,F–H). The effect of the bacterial endotoxin in MIA OCCs on *Il-1β* (*p* = 0.0001) and *Il-6* (*p* = 0.0014) expression was less distinct, whereas on *Cd206* (*p* = 0.0323), levels was more pronounced than that in control OCCs (Figure 6B,C,E). Furthermore, quetiapine preincubation upregulated the mRNA expression of *Cd206* (*p* = 0.0256) in MIA OCCs (Figure 6E).

### 3.6. The Impact of Quetiapine and CD200Fc on IL-6 and IL-10 Protein Levels in Control and MIA OCCs under Basal and LPS-Induced Conditions

**Figure 7:**
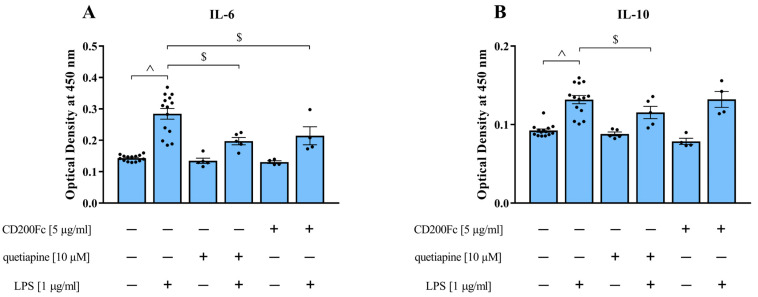
IL-6 (**A**) and IL-10 (**B**) protein levels in MIA OCCs after treatment with CD200Fc or quetiapine (10 µM) under basal and LPS-induced conditions. *n* = 4–14. The results were calculated as the optical density at 450 nm and are presented as the means ± standard errors of the means (SEM). Statistical analysis was performed using planned comparisons via a one-way ANOVA (contrast analysis). ^ *p* < 0.05 vs. MIA OCCs + vehicle, $ *p* < 0.05 vs. MIA OCCs + LPS.

In the final experiments, we attempted to investigate whether the limited response of MIA OCCs to quetiapine, and thus, its slight modulating effect on the gene expression of pro- and anti-inflammatory cytokines, was related to the changed susceptibility of this type of OCCs as a result of MIA. For this purpose, we compared the impact of the drug to CD200Fc for which anti-inflammatory properties have been suggested. We evaluated the protein levels of pro-inflammatory IL-6 and anti-inflammatory IL-10 after stimulation with CD200Fc or quetiapine under basal and LPS-induced conditions in MIA OCCs (Figure 7).

Treatment with the bacterial endotoxin elevated IL-6 production (*p* < 0.0001), which was reduced by both CD200Fc (*p* = 0.0050) and quetiapine (*p* = 0.0002) preincubation (Figure 7A). In the case of the IL-10 level, which was also increased in response to LPS exposure (*p* < 0.0001), the stimulation with the drug (*p* = 0.0381) diminished, while CD200Fc addition did not influence this disturbance (Figure 7B).

## 4. Discussion

In the present study, we demonstrated that MIA had an adverse effect on the biochemical status of OCCs and the expression of microglial receptors involved in neuron–microglia interactions. The changes were observed under basal conditions and/or after additional treatment with LPS applied in our research as a “second hit” activation. Specifically, MIA intensified the basal secretion of LDH and simultaneously NO release when the cultures were exposed to the bacterial endotoxin. Another important consequence of MIA was the strong limitation of *Cd200r* expression under nonstimulated conditions. The deficit was further enhanced by the introduction of the “second hit” to the experimental setup, which concurrently resulted in decreases of *Cx3cr1* levels in both types of OCCs. Nevertheless, the alterations in the gene expression of both receptors due to MIA were not followed by disturbances in the mRNA profile of the evaluated pro- and anti-inflammatory markers. Regarding quetiapine, although this atypical antipsychotic did not directly affect the expression of the ligand-receptor axes or the protein levels of CD200R and CX3CR1, drug administration upregulated *Cd206* expression and inhibited the induction of the “second hit” increase in IL-6 and IL-10 protein levels in MIA OCCs. The beneficial impact of quetiapine was also present in control OCCs, where the antipsychotic prevented *Il-1β*, *Il-6*, *Cebpb* and *Arg1* expression from rising after LPS treatment. Therefore, our observations suggest that MIA can influence the biochemical and immune profile of OCCs and determine the response to quetiapine treatment.

### 4.1. Maternal Immune Activation Affects Lactate Dehydrogenase Release in Organotypic Cortical Cultures and Partially Their Response to Stimulation with Lipopolysaccharide

MIA-generated behavioural, neurological and immunological alterations described in animals’ offspring at juvenile and adult stages of life are considered to reflect the aetiology of neuropsychiatric disorders in humans [42,43,44]. Nonetheless, the molecular mechanisms leading to these changes are still ambiguous, justifying a constant need for developing useful models to broaden the knowledge about these phenomena and for identifying new targets for potentially more effective pharmacotherapies.

Our present research was conducted in a neurodevelopmental model of schizophrenia based on the administration of bacterial endotoxin (specifically, LPS) to female rats throughout pregnancy [15,29,30,33,34,45]. MIA applied in this paradigm produces age-dependent behavioural deficits in sensorimotor gating [29,30], disturbances in exploration [33,45], spontaneous and amphetamine-induced locomotor activity [35] and social interactions [45] as well as the presence of anxiety behaviour [33]. Concurrently, MIA with LPS generates various long-lasting biochemical consequences, including peripheral and central immune disturbances [29,30,33,46,47]. Data have indicated that proper neuron–microglia communication is vital for the control of the immune response, whereas dysfunction of this dynamic crosstalk results in microglial activation and exaggerated inflammation [23,48,49]. In a proof-of-concept study, we revealed alterations in the CX3CL1-CX3CR1 and CD200-CD200R axes in the MIA model, and we have suggested that some of these can be essential for the development of behavioural disturbances [30].

As a consequence of the abovementioned data, the first primary objective of the present study was to establish whether MIA might also affect the basal and additionally stimulated by the bacterial endotoxin biochemical and immunological statuses of the offspring’s frontal cortex in ex vivo conditions. To answer this question, we employed organotypic cultures prepared from this brain structure, as its role in schizophrenia-like pathomechanisms is widely accepted [50,51,52]. The main advantages of organotypic cultures are their ability to partially retain a three-dimensional organization as well as the fact that they are anatomically intact and reflect, to some extent, their brain area of origin [53]. Since neurons and glia in OCCs represent the populations of corresponding cells found in vivo [54,55], this type of culture can constitute an alternative tool to certain in vivo experiments, allowing for the reduction in the number of animals required for studies [56] on cellular and molecular processes of the brain ex vivo [53].

In our study, we showed that both control and MIA OCCs were susceptible to LPS exposure, as confirmed by not only the higher secretion of LDH and NO but also the extensive immunological response (an increase in *Il-1β*, *Il-6*, *Cebpb*, *Arg1* and *Il-10* as well as a decrease in *Tgf-β* mRNA expression in both types of OCCs and additionally elevated *Cd40* level in control OCCs) to treatment with this bacterial endotoxin. Comparable observations regarding the effectiveness of LPS stimulation were described for control organotypic cultures prepared from the hippocampus (OHCs), in which incubation with the bacterial endotoxin resulted in the upregulated efflux of LDH, raised propidium iodide uptake, enhanced levels of nitrite, iNOS, IL-1β and TNF-α as well as decreased thiazolyl blue tetrazolium bromide reduction [57,58]. Moreover, in the present research, we demonstrated that MIA led to higher LDH release and increased vulnerability of OCCs to external stimulation with LPS, as revealed by the elevated secretion of NO. These results may indicate that MIA negatively affected the elementary biochemical processes of the cultures and intensified their susceptibility to the adverse influence of the additional stimulus. A similar phenomenon resembling the “priming” effect to subsequent LPS treatment was presented for OHCs prepared from the offspring of female rats with experimentally induced diabetes [59]. In this type of culture, incubation with the bacterial endotoxin caused a more pronounced inflammatory response and upregulated propidium iodide uptake than in control OHCs. All the abovementioned information further affirms the significance of the events occurring in the prenatal period on the developmental trajectory of the central nervous system and their possible long-lasting consequences for the offspring.

Gene expression analyses conducted herein showed that MIA significantly reduced the *Cd200r* mRNA level in OCCs. Exposure to the bacterial endotoxin (applied as a “second hit”) enhanced the impact of MIA and simultaneously triggered a deficit in *Cx3cr1* expression in both types of cultures. In previous studies, we revealed that MIA-generated malfunctions in the neuron–microglia axes were already present in the brains of the offspring at the early stages of life, specifically when rats were 7 days old [30]. Hence, our current data, corresponding partially with the observations obtained in the *in vivo* MIA model, seem to support the translational nature of the OCC technique.

In the present research, disturbances in gene expression were not accompanied by altered protein levels of CD200R or CX3CR1 in MIA OCCs under basal conditions or after additional LPS stimulation. The incompatible influence of MIA on these parameters might emerge from alterations in the regulation of various stages of mRNA expression, starting with changes in chromatin conformation, gene activation in response to external stimuli and control of the transcription process [60,61].

CD200-CD200R signalling constitutes one of the well-characterized checkpoint mechanisms that restrains the immune activity of microglia [62,63]. Through binding with the cognate receptor, CD200 initiates an intracellular signalling cascade, resulting in a general inhibition of microglial pro-inflammatory marker expression and cytokine responses to immune stimuli [64]. Malfunction of CD200-CD200R communication, as well as the CX3CL1-CX3CR1 pair, another critical neuron–microglia axis, limited the resolution of inflammation, potentiated microglial pro-inflammatory activity and exacerbated disease severity and/or progression in several models of neuroinflammatory diseases [15,65,66]. Herein, we detected deficits in *Cd200r* and/or *Cx3cr1* expression in MIA OCCs, yet we did not find significant changes in the mRNA levels of microglial markers (*Cd40*, *Cd206*, *Arg1* and *Cebpb*) or the expression profiles of pro- (*Il-1β* and *Il-6*) and anti-inflammatory (*Il-10* and *Tgf-β*) cytokines. Furthermore, the “second hit” did not particularly affect the microglial phenotype. In line with these observations, some researchers have suggested that disturbances in microglial activation are not a prerequisite for the presence of various deficits, including behavioural ones in MIA models [67,68].

### 4.2. Quetiapine Exerts Immunomodulatory Effects in Organotypic Cortical Cultures

In the second part of our study, we attempted to determine whether the anomalies in the expression of microglial receptors in MIA OCCs could represent a relevant target for quetiapine treatment. Unexpectedly, we did not observe an impact of quetiapine administration on the expression of the *Cd200*-*Cd200r* and *Cx3cl1*-*Cx3cr1* systems in either control or MIA OCCs. Concurrently, the downregulation of *Cd200r* and *Cx3cr1* gene expression resulting from the “second hit” with LPS was not modified by this drug. The lack of an effect of quetiapine treatment on neuron–microglia interactions was also displayed using ELISAs, quantifying the protein levels of both receptors in MIA OCCs.

At the mRNA level, the anti-inflammatory potential of quetiapine was presented in MIA OCCs in the form of the upregulation of *Cd206* expression. The key role of this mannose receptor, among others, is to regulate the secretion of molecules during the inflammatory response [69,70]. Therefore, generally, CD206 expression during inflammation occurs at low levels, while in the course of the resolution of inflammation, it increases to ensure the clearance of dismissed inflammatory factors [71,72].

Regarding the influence of quetiapine on cytokine release, the antipsychotic prevented the boost of IL-6 production in MIA OCCs after the additional stimulation with LPS. Comparatively, Grolli et al. [73] demonstrated the suppressive quetiapine potential on IL-6 synthesis in the serum of rats submitted to chronic stress. The regulation of IL-6-mediated processes is highly precise, with several intracellular pathways being involved [74,75]. IL-6 forms a system with a specific IL-6R and can bind with two glycoprotein 130 (gp130) subunits with high affinity, creating a tetramer or hexamer IL-6/IL-6R/gp130 complex [75]. The gp130 molecule conducts biological signals through two major pathways, including the JAK/STAT and Ras/MAPK signalling cascades. Among the JAK kinases, the role of JAK1 with its main substrate, transcription factor STAT3, is of particular significance in the inflammatory response [76]. Additionally, quetiapine can inhibit the activation of NFκB signalling in neurons [77,78] and glial cells, simultaneously reducing the release of IL-1β and TNF-α [22].

Exposure to bacterial endotoxins constitutes one of the most potent inducers of not only pro- but also anti-inflammatory factors, including IL-10. Generally, this cytokine plays a critical role in balancing immune responses to prevent chronic inflammatory diseases [79]. In our research, pretreatment with quetiapine diminished the production of this negative modulator of inflammation in MIA OCCs subjected to LPS stimulation. This observation corresponds to some extent with the findings described by Jaehne et al. [80], who revealed that both quetiapine and its primary active metabolite norquetiapine appeared to affect the increase in IL-10 expression noted at 24 h post incubation with the bacterial endotoxin. Therefore, the suppressive impact of quetiapine on IL-10 level is at least partially an adverse effect leading to prolongation of the inflammatory reaction and preventing a return to homeostasis after LPS treatment. Based on the literature data [81,82,83], the role of LPS-induced activation of common signalling pathways for IL-6 and IL-10, including the JAK/STAT system, may be crucial to this phenomenon. However, this hypothesis demands further confirmation.

Furthermore, in our study, the potential of quetiapine was displayed in control OCCs in the form of suppression of *Il-1β*, *Il-6*, *Cebpb* and *Arg1* expression under incubation with the bacterial endotoxin. To date, data concerning the anti-inflammatory action of quetiapine have varied and depended on the experimental model used, the dose and the administration method of the drug. Previously published reports underlined a double-edged immunomodulatory mechanism of this antipsychotic [15], which could be crucial in the context of the efficacy of quetiapine, as it was also influenced by the prior inflammatory activation state of cells [84]. Thus, the modulatory potential of quetiapine might be related to the inhibition of pro-inflammatory response rather than the stimulation of its anti-inflammatory components. Nevertheless, the mechanism of this action requires further research, particularly in ex vivo conditions.

To consolidate the results of the current research regarding the immunomodulatory properties of quetiapine and the detected deficiency in *Cd200r* expression in MIA OCCs, we introduced the positive regulation of CD200R via its agonist, specifically CD200Fc [85,86], to our experimental design. The notable finding described within this article is the suppressive effect of CD200Fc treatment on the IL-6 release in MIA OCCs in the presence of the “second hit”. The external modulation of CD200R via its ligand did not produce a more prominent impact on the investigated parameters in MIA OCCs than quetiapine stimulation. To the best of our knowledge, these results are the first that compared the action of quetiapine and CD200R modulation in MIA OCCs, providing new information on this phenomenon.

### 4.3. Limitations of the Study

We are fully aware of the limitations of the present research. First, only one dose of quetiapine (10 µM) was selected for the main experiments in our study, which undoubtedly reduced the basis for unambiguous conclusions regarding the mechanism of action of this antipsychotic. Second, we performed the analyses by applying only one selected concentration of LPS (1 µg/mL), the effect of which as the “second hit” stimulation was limited in some experiments and could subsequently influence the further effectiveness of quetiapine treatment. Moreover, in terms of most of the examined immunological parameters, we quantified only mRNA expression without measuring their protein levels. Furthermore, a broader perspective on the CD200-CD200R dyad would be achieved by additional investigation of the activation of signalling pathways related to this axis. Finally, the matter of high variability in group sizes including a couple of small groups in some experiments should be noted.

## 5. Conclusions

The present study demonstrated that MIA had an adverse effect on the biochemical status of OCCs and the expression of microglial receptors engaged in neuron–microglia communication under basal conditions and/or after “second hit” stimulation. The MIA procedure determined the OCCs’ response to quetiapine treatment, the immunomodulatory potential of which was expressed mainly as the inhibition of the negative impact of LPS on IL-6 and IL-10 release. In the current research, we did not observe the direct involvement of the CD200-CD200R axis in the mechanism of quetiapine action. Therefore, the question of whether the effects of CD200R modulation via CD200Fc and antipsychotic administration may be interrelated remains open, supporting further research on the involvement of this microglial receptor in the immunoregulating properties of quetiapine. Undoubtedly, our data strengthened the utility of the MIA model and OCC technique for investigating the processes underlying prenatal immune activation and searching for new targets for the pharmacological treatment of MIA-related conditions.

## Data Availability

All data supporting the conclusions of this manuscript are provided in the text and figures.
